# Socio-demographic predictors of not having private dental insurance coverage: machine-learning algorithms may help identify the disadvantaged

**DOI:** 10.1186/s12889-024-18868-1

**Published:** 2024-05-23

**Authors:** Venkata R. Duvvuri, Mona Abdelrehim, Sonica Singhal

**Affiliations:** 1 Public Health Ontario, Toronto, ON Canada; 2 Department of Laboratory Medicine and Pathobiology, Temerty Faculty of Medicine, University of Toronto, Toronto, ON Canada; 3 Dental Public Health, Faculty of Dentistry, University of Toronto, Toronto, ON Canada

**Keywords:** Access to dental care, Dental insurance, Dental care utilization, Barriers to dental care

## Abstract

**Background:**

For accessing dental care in Canada, approximately 62% of the population has employment-based insurance, 6% have some publicly funded coverage, and 32% have to pay out-of pocket. Those with no insurance or public coverage find dental care more unaffordable compared to those with private insurance. To support the development of more comprehensive publicly funded dental care programs, it is important to understand the socio-demographic attributes of all those, who find dental care unaffordable.

**Methods:**

This study is a secondary analysis of the data collected from Ontarians during the latest available cycle of the Canadian Community Health Survey (2017-18), a cross-sectional survey that collects information on health status, health care utilization, and health determinants for the Canadian population. First, bivariate analysis was conducted to determine the characteristics of Ontarians who lack dental insurance. Afterwards, we employed machine learning (ML) to analyze data and identify risk indicators for not having private dental insurance. Specifically, we trained several supervised ML models and utilized Shapley additive explanations (SHAP) to determine the relative feature importance for not having private dental insurance from the best ML model [the gradient boosting (GBM)].

**Results:**

Approximately one-third of Ontarians do not have private insurance coverage for dental care. Individuals with an income below $20,000, those unemployed or working part-time, seniors aged above 70, and those unable to afford to have their own housing are more at risk of not having private dental insurance, leading to financial barriers in accessing dental care.

**Conclusion:**

In the future, government-funded programs can incorporate these identified risk indicators when determining eligible populations for publicly funded dental programs. Understanding these attributes is critical for developing targeted and effective interventions, ensuring equitable access to dental care for Canadians.

**Supplementary Information:**

The online version contains supplementary material available at 10.1186/s12889-024-18868-1.

## Introduction

Canadians cover their dental expenses majorly in three ways: (1) 62% through private dental insurance (benefits from employer or purchased themselves); (2) 32% through out-of-pocket payments (paid at the point of care); and (3) 6% utilizing publicly funded targeted dental programs (federal/provincial/municipal). These public programs primarily target children (from low-income families), low-income seniors, eligible Indigenous individuals, people on social assistance, and those with disabilities [1, 2]. With many Canadians financing their own dental care through out-of-pocket expenses, cost becomes the predominant factor limiting access to care [3, 4]. Also, those covered through public programs, generally have limited coverage, which limits their treatment choices. Consequently, the current dental care financing system inequitably impacts those who need the care the most, a phenomenon of ‘inverse care law’ which has been observed among Canadian populations for accessing dental care [5]. Previous studies revealed that insurance and income are the strongest predictors of reporting cost barriers to receiving adequate dental care in Canada [6–9], where those with low-income and no insurance reported more cost barriers to accessing dental care compared to their counterparts. Moreover, the method of payment for dental care impacts its affordability, with high-income families primarily relying on private insurance and low-income households mainly paying out-of-pocket [10]. Therefore, the lack of affordable dental care and its negative implications could, in part, be addressed through interventions aimed at these two determinants.

Partly in response to this need, the federal government in 2022 has announced plans to establish a Canadian Dental Care Program (CDCP) for low-and middle-income Canadians [11]. The plan would provide coverage for uninsured Canadians with a household income of less than $90,000 a year with no co-pays for those earning under $70,000. By the end of 2023, the program will start covering those who are under 18 years old, persons with disabilities, and seniors, with full implementation by the end of 2025 to cover all from families, who are financially eligible. This plan will be funded with an investment of $13 billion over five years, starting in 2023-24, and $4.4 billion ongoing for implementation [12]. It is expected to support up to nine million uninsured Canadians once fully implemented. This initiative, which has tried to address both aspects, low income and no insurance, indeed would be the most significant health care initiative since Canadian Medicare was established [11].

Previous Canadian studies have categorized insurance status as follows: employment-based insurance, self-purchased insurance, government-based insurance, and no insurance. However, the upcoming Canadian Dental Care Plan simplifies this classification into two categories: insured individuals, who have any form of private insurance including employment-based or self-purchased, while uninsured are considered those, who do not have access to any form of private insurance; however, may be eligible for public insurance. Given that, it is important to know the socio-demographic attributes of those who do not have private dental insurance coverage. Understanding these risk attributes would provide an evidence-informed scientific basis to policymakers to assess the eligible population for this upcoming CDCP. Hence, this paper analyzes the Canadian Community Health Survey (CCHS) 2017-18 data using machine learning to identify the stronger predictors for not having private dental insurance.

## Materials and methods

Our study is a secondary data analysis of a cross-sectional national survey that covers 97% of the Canadian population, the CCHS (cycle: 2017-18, the latest cycle with oral health data of interest available). The CCHS gathers data on health status, healthcare utilization, and health determinants for the Canadian population. It targets individuals aged 12 and above residing in private dwellings across all 13 Canadian provinces and territories. However, it excludes individuals living on reserves, Indigenous settlements within provinces, full-time Canadian Forces members, and those in institutionalized settings from its sampling frame. The oral health and dental care questionnaires constitute optional content in the survey. This content was tailored to meet specific provincial-level requirements, leading to the inclusion of optional questions in select provinces during each survey cycle, with variations in their content. In the most recent cycle (2017-18), both oral health and dental care data were gathered for Ontario. For more details on the design and sampling features of the CCHS, please refer to the user guide [13].The Public Use Microdata Files (PUMF) for the 2017-18 CCHS data were accessed online using the Survey Documentation and Analysis (SDA) online tool available through the University of Toronto library at the Computing in the Humanities and Social Sciences (CHASS) portal. No ethics review was sought for the study, as this was a secondary data analysis of anonymized data that contained no personal identifiers, nor was it linked to any other data source [14].

### Study populations and variables (or features)

This study utilized data from the CCHS encompassing all health regions in Ontario, including East, West, Central, North, and Toronto, with a total sample size of 19,799. The dataset includes variables related to socio-demographics, oral health, general and mental health (Table S1). The dependent variable is “Type of Dental Insurance”, where we created a binary dependent variable where “having private dental insurance” (combining both employment-based and private dental insurance) and “not having private dental insurance” (combining government-based dental insurance and not having dental insurance) to achieve the study goal: to identify the population characteristics of not having private dental insurance. The individuals with government-based insurance and those without any insurance were grouped together as “not having private dental insurance” due to the specific socio-economic context in Ontario. In the current circumstances in Ontario, as these data are only from Ontario, the low income cut-off for being on public programs is very low, for example, for Healthy Smiles Ontario, it is $28.560 family income for a family with two children. A lot of Ontarians, with low- and middle-income do not have private insurance, as they are working on contract casual positions, or part-time, however do not qualify for public programs as they do not meet the income threshold. This has been recognized and therefore the upcoming Canadian Dental Care Plan has income eligibility of annual family net income of $90,000 or less. In addition, people who have public insurance or no insurance qualify for this program but not those, who have private insurance [11, 15].

### Data pre-processing, and feature engineering

Table S1 presents a list of independent variables, related to socio-demographics, oral health, general and mental health, and the dependent variable before and after pre-processing and feature engineering. We used 16 socio-demographic, 12 oral health, 4 general or mental health and one dental insurance variable are used in the subsequent analysis, as indicated in the last column of Table S1.

### Machine learning: feature selection, modelling, analysis and evaluation

To prepare the dataset for analysis, categorical variables were transformed into dummy variables, a process also known as one-hot encoding that resulted cleaned dataset (dimensions 11,877 × 53 variables). Variance Inflation Factor (VIF) analysis was conducted as part of feature selection to detect the multicollinearity amongst the independent variables, where a VIF ≥ 5 indicates potential problematic levels of multicollinearity and VIF ≥ 10 indicates extreme multicollinearity (Figure S1) [16]. The total dataset was split into 80% (*n* = 15,839) was allocated to training with 10-fold cross validation and 20% (*n* = 3960) was allocated to testing. The dependent variable to predict is “type of dental insurance” which was coded as binary: “having private dental insurance” (combining both employment-based and private dental insurance) and “not having private dental insurance”. Before building ML models, it is critical to assess whether the training dataset exhibits class imbalance stemming from sub-categories within the dependent variable. Class imbalance can significantly affect the dependability, equity, and efficacy of ML models, potentially leading to misclassification of minority classes due to a bias towards the majority class. In our dataset, we noticed class imbalance where “having private dental insurance” (*n* = 12,710) and “not having private dental insurance” (*n* = 7089). To treat class imbalance, we implemented the following commonly used data-level resampling techniques, Oversampling (increase the number of instances in the minority class, not having private dental insurance), Under-sampling (decrease the number of instances in the majority class, having private dental insurance) and Synthetic Minority Over-sampling Technique (SMOTE, generates synthetic samples for the minority class, not having private dental insurance) [17]. This will result in total of four datasets (three datasets from resampling techniques, and one original dataset).

Python v3.9.17 and packages scikit-learn 1.4.1 were used to build supervised ML models which ranged from commonly used classifiers including logistic regression (LR), penalized LR with least absolute shrinkage and selection operator LASSO (LR-LASSO), LR with RIDGE (LR-RIDGE), decision tree (DTREE), random forest (RF), adaptive boosting (ADB), bootstrap aggregating (BAG), gradient boosting (GBM), and extreme gradient boosting (XGBT v1.7.3). Due to the presence of class imbalance and the consequences of undermining the false negatives, the recall (sensitivity) metric was chosen to identify the best ML classifiers on the training datasets. This best ML classifier with each training dataset was further examined for its performance and conducted hyper-parameter tuning or optimization (the process of selecting the optimal hyper-parameters for a ML algorithm to maximize its performance on a given dataset). The models’ performance was evaluated by assessing accuracy, precision, recall, F1-score, and the area under the receiver operating characteristic (AUROC) [18]. Finally, the Shapley Additive Explanations (SHAP, v0.44.1) package was used to interpret the impact of top discriminatory variables on the model with the highest relative performance. SHAP uses cooperative game theory to calculate the marginal contribution of each feature and examines the feature influence on model prediction [19]. Scikit-learn v0.24.2, a popular Python library for data science and machine learning tasks was implemented.

### Statistical analysis

All categorical variables were presented as numbers and percentages. The statistical significance was calculated using Pearson’s Chi-square test. R package, Arsenal v3.2.7 was used to prepare large-scale statistical summaries i.e., Table 1.

## Results

### Baseline characteristics of the study populations

Table 1 shows the baseline characteristics of Ontarians with and without private dental insurance in the 2017-18 CCHS. In our paper, private dental insurance includes employment-based and self-purchased insurance. Overall, 35.8% of Ontarians do not have private insurance; however, stratified by varied socio-demographic attributes, proportions are revealed quite differently. By income, 83.8% of Ontarians with household annual incomes below $20,000, and 64.4% of those below $40,000, have no private dental insurance. By education, 71.4% of those having less than secondary education, and by age, 61.1% of individuals aged 70–79 years, do not have private insurance. Additionally, more than 50% of those who were unemployed, housing in a rented accommodation, and perceived their oral or general health as “fair” to “poor” had no private dental insurance.

**Table 1:** Baseline characteristics of CCHS data (2017-18)

Independent Variables	Dependent variable		Total (*N* = 19,799)
	Having private dental insurance **(** ***N*** ** = 12,710) (64.2%)**	Not having private dental insurance **(** ***N*** **( = 7089) (35.8%)**	
**Age**			
12–19 years	621 (73.0%)	230 (27.0%)	851
20–29 years	1414 (61.7%)	879 (38.3%)	2293
30–39 years	2407 (70.8%)	993 (29.2%)	3400
40–49 years	2528 (76.0%)	797 (24.0%)	3325
50–59 years	2813 (69.8%)	1217 (30.2%)	4030
60–69 years	2279 (53.8%)	1955 (46.2%)	4234
70–79 years	648 (38.9%)	1018 (61.1%)	1666
**Sex**			
Female	6886 (63.2%)	4017 (36.8%)	10,903
Male	5824 (65.5%)	3072 (34.5%)	8896
**Sexual orientation**			
Heterosexual	12,394 (64.4%)	6854 (35.6%)	19,248
Homosexual or bisexual	316 (57.4%)	235 (42.6%)	551
**Household income**			
No income to $19,999	226 (16.2%)	1167 (83.8%)	1393
$20,000 to $39,999	785 (32.6%)	1626 (67.4%)	2411
$40,000 to $59,999	1555 (54.1%)	1321 (45.9%)	2876
$60,000 to $79,999	1748 (65.8%)	908 (34.2%)	2656
>=$80,000	8396 (80.2%)	2067 (19.8%)	10,463
**Household Education**			
Less than secondary school	257 (28.6%)	643 (71.4%)	900
More than secondary school	12,453 (65.9%)	6446 (34.1%)	18,899
**Employment status**			
Full-time	8842 (76.8%)	2677 (23.2%)	11,519
Part-time	1304 (57.7%)	955 (42.3%)	2259
Unemployed	2564 (42.6%)	3457 (57.4%)	6021
**Attending school or university**			
No	11,668 (63.8%)	6620 (36.2%)	18,288
Yes	1042 (69.0%)	469 (31.0%)	1511
**Marital status**			
Married / common-law	8168 (70.9%)	3359 (29.1%)	11,527
Single	2823 (58.4%)	2012 (41.6%)	4835
Widowed/ divorced/ separated	1719 (50.0%)	1718 (50.0%)	3437
**Geographical Location**			
Central Ontario	2794 (67.4%)	1351 (32.6%)	4145
East Ontario	3155 (64.8%)	1716 (35.2%)	4871
Northern Ontario	1570 (63.6%)	899 (36.4%)	2469
Toronto	852 (61.3%)	537 (38.7%)	1389
Western Ontario	4339 (62.7%)	2586 (37.3%)	6925
**Dwelling ownership**			
Owned	10,584 (69.4%)	4671 (30.6%)	15,255
Rented	2126 (46.8%)	2418 (53.2%)	4544
**Household size**			
More than three	5476 (72.0%)	2132 (28.0%)	7608
Two	4563 (63.6%)	2609 (36.4%)	7172
One	2671 (53.2%)	2348 (46.8%)	5019
**Number of < 5 year old in the household**			
More than one	1689 (71.3%)	680 (28.7%)	2369
No	11,021 (63.2%)	6409 (36.8%)	17,430
**Country of birth**			
Canada	10,117 (65.6%)	5299 (34.4%)	15,416
Other	2593 (59.2%)	1790 (40.8%)	4383
**Cultural/racial background**			
Non-white	1969 (62.3%)	1190 (37.7%)	3159
white	10,741 (64.5%)	5899 (35.5%)	16,640
**Languages spoken most often at home**			
English	11,479 (65.0%)	6191 (35.0%)	17,670
English and French	384 (68.1%)	180 (31.9%)	564
French	253 (64.7%)	138 (35.3%)	391
Other	594 (50.6%)	580 (49.4%)	1174
**Last time visited a dental professional**			
More than one year	1675 (40.8%)	2432 (59.2%)	4107
One year of less	11,035 (70.3%)	4657 (29.7%)	15,692
**Avoided visiting a dental professional due to cost within 12 months**			
No	11,355 (73.8%)	4040 (26.2%)	15,395
Yes	1355 (30.8%)	3049 (69.2%)	4404
**Having dentures or false teeth**			
No	11,508 (66.2%)	5874 (33.8%)	17,382
Yes	1202 (49.7%)	1215 (50.3%)	2417
**Perceived oral health**			
Fair to poor	1027 (46.2%)	1197 (53.8%)	2224
Good to excellent	11,683 (66.5%)	5892 (33.5%)	17,575
**Satisfaction with teeth**			
Dissatisfied to very dissatisfied	755 (46.4%)	872 (53.6%)	1627
Neither satisfied nor dissatisfied	1323 (61.1%)	844 (38.9%)	2167
Satisfied to very satisfied	10,632 (66.4%)	5373 (33.6%)	16,005
**Problems with mouth uncomfortable to eat food**			
Often or sometimes	1719 (54.7%)	1425 (45.3%)	3144
Rarely or never	10,991 (66.0%)	5664 (34.0%)	16,655
**Problems with mouth to avoid particular foods**			
Often or sometimes	1107 (50.2%)	1099 (49.8%)	2206
Rarely or never	11,603 (66.0%)	5990 (34.0%)	17,593
**Problems with mouth and other persistent pain**			
Often or sometimes	1325 (55.9%)	1044 (44.1%)	2369
Rarely or never	11,385 (65.3%)	6045 (34.7%)	17,430
**Had bleeding gums**			
Often or sometimes	3002 (63.7%)	1714 (36.3%)	4716
Rarely or never	9708 (64.4%)	5375 (35.6%)	15,083
**Had persistent bad breath**			
Often or sometimes	1649 (57.1%)	1237 (42.9%)	2886
Rarely or never	11,061 (65.4%)	5852 (34.6%)	16,913
**Perceived general health**			
Fair to poor	992 (46.1%)	1159 (53.9%)	2151
Good to excellent	11,718 (66.4%)	5930 (33.6%)	17,648
**Perceived mental health**			
Fair to poor	804 (50.8%)	779 (49.2%)	1583
Good to excellent	11,906 (65.4%)	6310 (34.6%)	18,216
**Perceived life stress**			
No	4268 (60.3%)	2809 (39.7%)	7077
Yes	8442 (66.4%)	4280 (33.6%)	12,722
Strong	9148 (65.0%)	4919 (35.0%)	14,067
Weak	3562 (62.1%)	2170 (37.9%)	5732
Less than two times	2202 (57.9%)	1603 (42.1%)	3805
Two or more times	10,508 (65.7%)	5486 (34.3%)	15,994
Dissatisfied to very dissatisfied	225 (39.1%)	351 (60.9%)	576
Neither satisfied nor dissatisfied	374 (43.4%)	488 (56.6%)	862
Satisfied to very satisfied	12,111 (66.0%)	6250 (34.0%)	18,361

Risk model analysis and evaluation

Nine ML algorithms were deployed and evaluated for their ability to distinguish between having private dental insurance and not having private dental insurance, using the original dataset and three additional datasets generated through resampling techniques (Figure S2). The highest performing ML classifiers, as determined by the recall (sensitivity) metric across the four training datasets, were as follows: Random Forest with the original dataset (recall: 53.88%), Random Forest with the SMOTE dataset (recall: 84.07%), Random Forest with the Oversampling dataset (recall: 89.31%), and Gradient Boosting with the under-sampling dataset (recall: 74.30%). These models were further optimized by selecting optimal hyper-parameters to maximize each model’s performance on a given dataset.

Model evaluation metrics of these ML models were measured to identify the most effective model based on performance (Table 2). The GBM model, trained on the under-sampling dataset, demonstrated superior performance in reducing false negatives, as evidenced by its high training recall of 0.7702 and test recall of 0.7522. Additionally, the model’s training accuracy of 0.7708 and test accuracy of 0.7513 indicate a well-balanced model with no significant over-fitting or under-fitting issues. An important observation is that LR, LR-LASSO and LR-RIDGE models with three resampled datasets performed well next to GBM. All models did not perform well on the original dataset, attributed to the inherent class imbalance.

**Table 2:** Selection of Optimal machine learning models based on the model evaluation matrices

Model and Dataset	Training Accuracy	Test Accuracy	Training Precision	Test Precision	Training Recall	Test Recall	Training F1	Test F1	Training ROCAUC	Test ROCAUC	Interpretation
GBM with Undersampling Data	0.7708	0.7513	0.772	0.7497	0.7684	0.7547	0.7702	0.7522	0.8548	0.8307	Best optimal model
LR-LASSO with Undersampling Data	0.7574	0.7413	0.7648	0.7401	0.7434	0.744	0.754	0.742	0.8367	0.8294	optimal model
LR-RIDGE with Undersampling Data	0.7574	0.7413	0.7648	0.744	0.7434	0.744	0.754	0.742	0.8367	0.8294	optimal model
LR- with Undersampling Data	0.7574	0.7413	0.7648	0.744	0.7434	0.744	0.754	0.742	0.8367	0.8294	optimal model
LR-RIDGE with Oversampling Data	0.7548	0.7491	0.7614	0.7512	0.7422	0.7448	0.7516	0.748	0.8344	0.8282	optimal model
LR with Oversampling Data	0.7548	0.7491	0.7614	0.7512	0.7422	0.7448	0.7516	0.748	0.8344	0.8282	optimal model
LR-LASSO with Oversampling Data	0.7552	0.7472	0.7641	0.7531	0.7382	0.7355	0.7509	0.7442	0.8359	0.83	optimal model
LR-LASSO with SMOTE Data	0.7992	0.822	0.8704	0.8774	0.7031	0.7485	0.7779	0.8078	0.8925	0.901	optimal model
LR-RIDGE with SMOTE Data	0.7245	0.7168	0.7455	0.7339	0.6816	0.6802	0.7121	0.706	0.7373	0.7272	optimal model
LR with SMOTE Data	0.7245	0.7168	0.7455	0.7339	0.6816	0.6802	0.7121	0.706	0.7373	0.7272	optimal model
RF with Oversampling Data	0.9749	0.6845	0.9723	0.7568	0.9776	0.5437	0.9749	0.6328	0.9983	0.7581	Overfitting*
RF with SMOTE Data	0.9721	0.7977	0.9833	0.8248	0.9605	0.7558	0.9718	0.7888	0.998	0.8713	Overfitting*
RF with Original Data	0.8243	0.7866	0.8268	0.7332	0.5606	0.5093	0.6681	0.6011	0.8724	0.8294	Overfitting*
LR with Original Data	0.7871	0.7946	0.719	0.7306	0.5335	0.5533	0.6125	0.6297	0.8352	0.8326	high false negatives based on the low recall
LR-RIDGE with Original Data	0.7871	0.7946	0.719	0.7306	0.5335	0.5533	0.6125	0.6297	0.8352	0.8326	high false negatives based on the low recall
LR-LASSO with Original Data	0.7868	0.7946	0.7184	0.7306	0.5329	0.5533	0.6119	0.6297	0.8352	0.8327	high false negatives based on the low recall

**Overfitting**: occurs when a model learns the detail and noise in the training data to the extent that it negatively impacts the model’s performance on new data. The model performs exceptionally well on the training data but poorly on the test data or new, unseen data

Feature importance (Risk attributes).

**Fig. 1:**
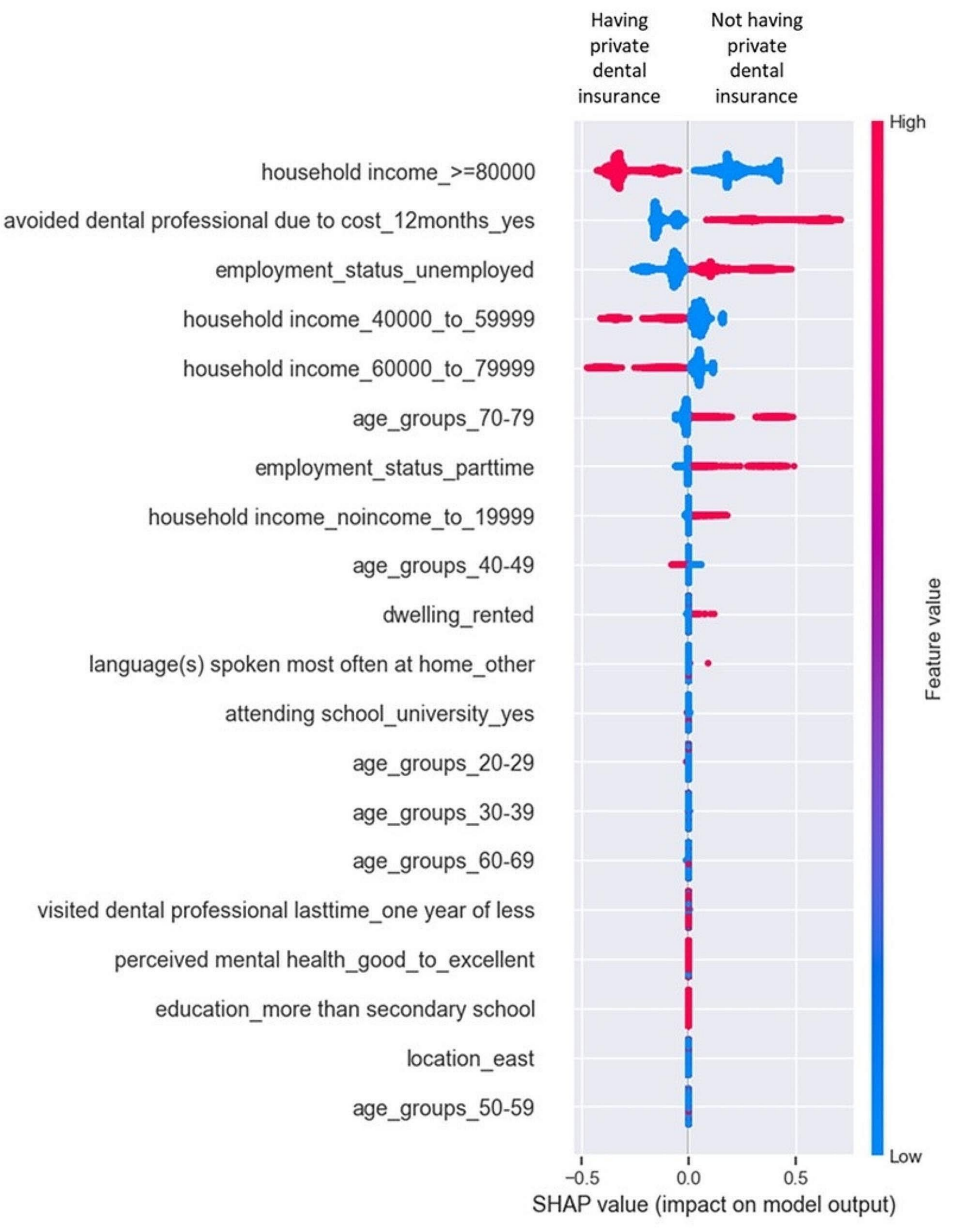
Gradient Boosting Model (GBM) - feature importance for having private dental insurance and not having private dental insurance. Shapley additive explanations (SHAP) was used to determine the relative feature importance for having private dental insurance and not having private dental insurance

Shapley additive explanations (SHAP) was used to determine the relative feature importance for having private dental insurance and not having private dental insurance from the best ML model (i.e., GBM). The SHAP summary plot in Fig. 1 combines feature importance with the feature effects on the model. The figure presents feature importance scores to determine the relative importance of each attribute in predicting the risk of not having dental insurance. The blue-red coloured bar presents the impact of feature value on the model. The attributes predicting higher risk are marked red while those of low risk are marked in the blue colour. As per the model, the attributes identified to be associated with not having private dental insurance include: ‘household income < 20,000’, ‘avoided dental professional due to cost within last 12 months’, ‘unemployment status’, ‘part-time employment’, ‘patient’s age-group > = 70–79’, and ‘dwelling rented.’

## Discussion

Using data from the latest available cycle of the CCHS (2017-18), this study identified population’s attributes for not having private dental insurance in Ontario. As the Canadian health care system excludes dental care, except for surgical dental procedures performed in hospital settings, Canada faces inequitable utilization of the oral health care system [20]. It is well established that low income and lack of insurance coverage play a crucial role in limiting people’s ability to access oral health care [7, 8, 21]. Additionally, literature on the subject has pointed out that although income and insurance are positively correlated, insurance has an independent effect on dental care utilization [22]. Regardless of income level, insured individuals are more likely to utilize and have better access to dental services than their uninsured counterparts [8, 10]. Previous studies have reported that poor access to dental care has a negative impact on individuals’ health, health care system and society [23–27]. Therefore, with 36% of the population lacking private dental insurance (including employment-based and self-purchased plans), it is important to identify what are the attributes of those disadvantaged population. This would provide policymakers with data-driven evidence on who should be included in the upcoming CDCP. By prioritizing the most vulnerable groups, existing oral health inequities can be expected to be addressed, thereby achieving better oral health for all.

In our study, we employed machine learning to analyze data and identify predictors for not having private dental insurance. Machine learning offers a robust and systematic approach to extracting patterns, relationships, and insights from complex datasets. Based on our findings, the GBM algorithm, in combination with under-sampling techniques, proved to be the most effective method for identifying predictors related to not having private dental insurance. This combination exhibited remarkable performance metrics, showcasing its proficiency in this context. The GBM algorithm, coupled with under-sampling, demonstrated impressive performance metrics (Table 2). This suggests not only effective handling of class imbalance but also a good generalization to unseen data without over-fitting or under-fitting. The evaluation of machine learning models on the original and three resampled datasets highlights the importance of choosing the right data preprocessing technique in conjunction with the model type.

In our ML model, the most significant variable for not having dental insurance was the cost barriers to dental care, followed by unemployment. It’s crucial to acknowledge the limitations of this cross-sectional study, as we cannot definitively determine whether cost-barriers to dental care is a risk indicator for not having dental insurance or if it is the other way around, where not having dental insurance is a risk indicator for avoiding dental visits due to financial barriers. Nevertheless, our results confirm a strong correlation between these two attributes. In Canada, employment-based plans constitute the majority of private dental insurance [28]. Employers offer non-wage benefits such as insurance plans to enhance the employer-employee contract; this offer is voluntary not obligatory [29]. Unemployment signifies the absence of workplace benefits, or the inability to self-purchase plans, resulting in the lack of private dental insurance. Additionally, part-time employment was a predictor in reporting no dental insurance. Part-time employment is defined as working less than 30 h per week, typically with lower hourly rates than their full-time counterparts [30, 31]. Employers as such offer dental benefits more to their permanent full-time employees than those who work on contract or part-time [32].

The third strongest predictor for having no insurance was being 70–79 years old; this might be explained by the fact that retired people lose their work benefits, particularly the employment-sponsored insurance. Additionally, retirees often rely on a limited fixed pension, restricting their ability to purchase private plans [33].

The fourth and fifth strongest predictors in our model are low income (less than $20,000/ year) and renting a house. Studies indicate that some low-wage workers remain uninsured, even when eligible for employer-based coverage, as they prioritize more pay in lieu of health benefits to cover other expenses [34, 35]. Furthermore, lack of home ownership status reflects housing insecurity that may affect the affordability of purchasing private dental insurance [36].

Our study has some methodological and study design limitations. It is a secondary data analysis of a national survey, thereby precluding the detection or correction of data entry errors from the original survey. Additionally, the CCHS is a cross-sectional survey and only associations can be assessed and no causal relationships can be inferred from this study. Further, the CCHS excluded individuals living on reserves and other Indigenous settlements in the provinces as well as the institutionalized population, potentially leading to underestimated findings and limited generalizability. Finally, potential measurement errors might have been introduced by respondent recall errors, inconsistency of their opinion, and the respondents’ tendency to provide socially desirable answers. That said, it is a generalized limitation of such surveys and studies based on such surveys. At the same time, using such large population-based data gives strength to the study. A substantial sample size, enabled us to make population-level estimations in Ontario. Moreover, the study employs machine learning to offer data-driven evidence to policymakers regarding vulnerable groups that need to be included in the upcoming national dental care plan.

## Conclusions

Approximately, one-third of Canadians do not have private insurance coverage for utilizing dental care. People with annual income of less than $20,000; are unemployed or having part-time employment; seniors above 70 years of age; and those, who are not able to afford their own house are more at risk of not having dental insurance and thereby face cost barriers to access dental care. Future government funded programs need to take into consideration these attributes when deciding the target populations eligible for publicly funded dental programs to ultimately address existing inequities in the Canadian oral health care system. Learning of these attributes can be helpful for other Organization for Economic Co-operation and Development countries as well when assessing eligible populations for publicly funded dental care programs. Also, this study underscores the complexity of model selection and the impact of data preprocessing techniques on machine learning performance. It highlights the necessity for careful consideration of model-data compatibility to achieve optimal performance and reliable predictive capabilities.

### Electronic supplementary material

Below is the link to the electronic supplementary material.

Supplementary Material 1

Supplementary Material 2

Supplementary Material 3

## Data Availability

The Public Use Microdata Files (PUMF) for the 2017-18 CCHS data were accessed online using the Survey Documentation and Analysis (SDA) online tool available through the University of Toronto library at the Computing in the Humanities and Social Sciences (CHASS) portal. Requests and further information on accessing the dataset can be obtained here: https://mdl.library.utoronto.ca/research/help.

## Abbreviations

CDCP: Canada Dental Care Plan
ML: Machine learning
CCHS: Canadian Community Health Survey
CHASS: Computing in the Humanities and Social Sciences
PUMF: Public Use Microdata Files
SMOTE: Synthetic minority oversampling technique
LR: Logistic regression
LASSO: Least absolute shrinkage and selection operator
RF: Random forest
GBM: Gradient boosting
AdaBoost: Adaptive boosting
SHAP: Shapley additive explanations

## References

[1] Canadian Dental Association. CDA responds to federal dental care announcement [Internet]. Ottawa, Ontario: Canadian Dental Association; 2022 [cited 2022 Dec 23]. https://www.cda-adc.ca/EN/about/media_room/news_releases/2022/09_13_federal_announcement.asp.

[2] Canadian Association of Public Health Dentistry. Government dental programs [Internet]. Canada: Canadian Association of Public Health Dentistry; 2022 [cited 2022 Dec 23]. https://caphd.ca/programs-resources/government-dental-programs/.

[3] Ramraj C, Quiñonez CR (2013). Self-reported cost-prohibitive dental care needs among canadians. Int J Dent Hyg.

[4] Ravaghi V, Quiñonez C, Allison PJ (2013). The magnitude of oral health inequalities in Canada: findings of the Canadian health measures survey. Commun Dent Oral Epidemiol.

[5] Dehmoobadsharifabadi A, Singhal S, Quiñonez C (2016). Investigating the inverse care law in dental care: a comparative analysis of Canadian jurisdictions. Can J Public Health.

[6] Thompson B, Cooney P, Lawrence H, Ravaghi V, Quiñonez C (2014). Cost as a barrier to accessing dental care: findings from a Canadian population-based study. J Public Health Dent.

[7] Bhatti T, Rana Z, Grootendorst P, Dental, Insurance. Income and the Use of Dental Care in Canada [Internet]. Vol. 73. 2007. www.cda-adc.ca/jcda. 17295945

[8] Millar WJ, Locker D (1999). Dental insurance and use of dental services. HEALTH REPORTS-STATISTICS Can.

[9] Abdelrehim M, Ravaghi V, Quiñonez C, Singhal S (2023). Trends in self-reported cost barriers to dental care in Ontario. PLoS ONE.

[10] Locker D, Maggirias J, Quiñonez C (2011). Income, dental insurance coverage, and financial barriers to dental care among Canadian adults. J Public Health Dent.

[11] Government of Canada. Making dental care more affordable: the Canadian Dental Benefit [Internet]. Ottawa, Ontario: Government of Canada; 2022 [cited 2022 Dec 23]. https://www.canada.ca/en/department-finance/news/2022/09/making-dental-care-more-affordable-the-canada-dental-benefit.html.

[12] Government of Canada. Health Canada. The Honourable Jean-Yves Duclos highlights budget investments for the Canadian Dental Care Plan. https://www.canada.ca/en/health-canada/news/2023/04/the-honourable-jean-yves-duclos-highlights-budget-investments-for-the-canadian-dental-care-plan0.html.

[13] Statistics Canada. Canadian Community Health Survey (CCHS) Annual component. User guide 2018 and 2017–2018 Microdata file. At:https://sda-artsci-utoronto-ca.myaccess.library.utoronto.ca/legacy_sda/dli2/cchs/cchs2017/more_doc/CCHS%202017-2018%20User%20Guide.pdf.

[14] Canadian Institutes of Health Research, Natural Sciences and Engineering Research Council of Canada, & Social Sciences and Humanities Research Council of Canada. Tri-Council Policy Statement Ethical Conduct for Research Involving Humans 2018, p. 17. 211 p.

[15] Ontario. The Ministry of Health. Teeth cleaning, check-ups and dental treatment for kids. At:https://www.ontario.ca/page/get-dental-care.

[16] Vatcheva KP, Lee M, McCormick JB, Rahbar MH. Multicollinearity in regression analyses conducted in epidemiologic studies. Epidemiol (Sunnyvale Calif). 2016;6(2). 10.4172/2161-1165.1000227 PMC4888898 27274911

[17] Krawczyk B (2016). Learning from imbalanced data: open challenges and future directions. Progress Artif Intell.

[18] Hicks SA, Strümke I, Thambawita V, Hammou M, Riegler MA, Halvorsen P, Parasa S (2022). On evaluation metrics for medical applications of artificial intelligence. Sci Rep.

[19] Lundberg SM, Erion G, Chen H, DeGrave A, Prutkin JM, Nair B, Katz R, Himmelfarb J, Bansal N, Lee SI (2020). From local explanations to Global understanding with explainable AI for trees. Nat Mach Intell.

[20] Ravaghi V, Farmer J, Quiñonez C (2020). Persistent but narrowing oral health care inequalities in Canada from 2001 through 2016. J Am Dent Assoc.

[21] Zangiabadi S, Costanian C, Tamim H. Dental care use in Ontario: the Canadian community health survey (CCHS). BMC Oral Health. 2017;17(1). 10.1186/s12903-017-0453-7 PMC5747094 29284491

[22] Zivkovic N, Aldossri M, Gomaa N, Farmer JW, Singhal S, Quiñonez C et al. Providing dental insurance can positively impact oral health outcomes in Ontario. BMC Health Serv Res. 2020;20(1). 10.1186/s12913-020-4967-3 PMC7027064 32066434

[23] Hall-Scullin E, Whitehead H, Milsom K, Tickle M, Su TL, Walsh T (2017). Longitudinal study of Caries Development from Childhood to Adolescence. J Dent Res.

[24] Sheiham A (2006). Dental caries affects body weight, growth and quality of life in pre-school children. Br Dent J.

[25] Gil-Montoya JA, de Mello ALF, Barrios R, Gonzalez-Moles MA, Bravo M. Oral health in the elderly patient and its impact on general well-being: a nonsystematic review. Clinical Interventions in Aging. Volume 10. Dove Medical Press Ltd.; 2015. pp. 461–7. 10.2147/CIA.S54630 PMC4334280 25709420

[26] The Canadian Institute of Health Information. Treatment of preventable dental cavities in preschoolers: a focus on day surgery under general anesthesia. CIHI.2013:1–34.

[27] Hayes A, Azarpazhooh A, Dempster L, Ravaghi V, Quiñonez C. Time loss due to dental problems and treatment in the Canadian population: analysis of a nationwide cross-sectional survey [Internet]. 2013. http://www.biomedcentral.com/1472-6831/13/17. 10.1186/1472-6831-13-17 PMC3641013 23587069

[28] Abdelrehim M. Trends in self-reported cost barriers to dental care in Ontario [dissertation]. University of Toronto. Canada; 2022. 10.1371/journal.pone.0280370 PMC10328358 37418457

[29] Sano Y. Oral health, dental insur al health, dental insurance co ance coverage, and pr age, and preventive dental car e dental care utilization: The case of immigrants in Canada [dissertation]. University of Western Ontario. Canada; 2018.

[30] Statistics Canada. Tables 14-10-0340-01 Employee wages by occupation, annual. 10.25318/1410034001-eng.

[31] The Canadian Council on Social Development. Stata and Facts: Labour Market-Employment. At: http://www.ccsd.ca/factsheets/labour_market/employment/ccsd_labour_market_employment.pdf.

[32] Sadeghi L. Trends in Access to Dental Care among Middle-Class canadians. University of Toronto (Canada); 2012.

[33] Kelsall D, O’Keefe J (2014). Good health requires a healthy mouth: improving the oral health of Canada’s seniors. CMAJ.

[34] Royalty AB, Hagens J (2005). The effect of premiums on the decision to participate in health insurance and other fringe benefits offered by the employer: evidence from a real-world experiment. J Health Econ.

[35] Abraham JM, Vogt WB, Gaynor MS (2006). How do households choose their employer-based health insurance?. INQUIRY: J Health Care Organ Provis Financing.

[36] Giannoni M, Grignon M. Food insecurity, home ownership and income-related equity in dental care use and access: the case of Canada. BMC Public Health. 2022;22(1). 10.1186/s12889-022-12760-6 PMC8919598 35287642

