# Supplementary material for: Socio-demographic predictors of not having private dental insurance coverage: machine-learning algorithms may help identify the disadvantaged
Source: BMC Public Health. 2024 May 23;24:1386. doi: 10.1186/s12889-024-18868-1 (PMC11112852; doi:10.1186/s12889-024-18868-1)
Supplement: Supplementary file 1 — Supplementary Material 1 [file 12889_2024_18868_MOESM1_ESM.pdf]

Table S1: Summary of the variables used in this study.

CCHS code and name of the variable	Sub-categories of each variable	Renaming of independent variable or features (after feature engineering)	Final list of variables (sub-categories)	Variables considered in this study
**1. Socio-demographic variables**				
**Dhhgage**	1, 2, 3, 4, 5, 6, 7, 8, 9, 10, 11, 12, 13, 14, 15, 16	Age-groups	1. 12-19 years<br>2. 20-29 years<br>3. 30-39 years<br>4. 40-49 years<br>5. 50-59 years<br>6. 60-69 years<br>7. 70-79 years<br>8. 80 and older	Yes
**dhh_sex**<br>Sex	1,2	Sex	1=male<br>2=female	Yes
**dhhgms**<br>Marital status	1 = married<br>2= common-law<br>3= widowed/divorced/separated<br>4= single	Marital Status	1= married/common-law<br>2= widowed/divorced/separated<br>3= single	Yes
**sdcdgcb**<br>Country of birth-grouped	1= Canada<br>2= other	Country of birth	1= Canada<br>2= other	Yes
**incdghh**<br>Total household income-all sources	1= No income or less than $20,000<br>2= $20,000 to $39,999<br>3= $40,000 to $59,999<br>4= $60,000 to $79,999<br>5= $80,000 or more	Total household income	1= No income or less than $20,000<br>2= $20,000 to $39,999<br>3= $40,000 to $59,999<br>4= $60,000 to $79,999<br>5= $80,000 or more	Yes
**dhhdghsz**<br>Household size	1= one person living in household<br>2= two persons living in household<br>3= three persons living in household<br>4= four persons living in household<br>5= Grouped - 5 or more persons live in the household	Household size	1= One person living in household<br>2= Two persons living in household<br>3= Three or more persons living in household	Yes

**dhhdgle5**<br>Number of persons 5 years old or less in household	0= No persons under 6 in the household<br>1= One or more persons under 6 in the household		0= No persons under 6 in the household<br>1= One or more persons under 6 in the household	Yes
**sdc_035**<br>Considers self heterosexual / homosexual / bisexual	1= Heterosexual<br>2= Homosexual, that is lesbian or gay<br>3= bisexual		1= Heterosexual<br>2= Homosexual + bisexual	Yes
**mac_015**<br>Currently attending school/college/CEGEP/university	1= yes<br>2= no		1= yes<br>2= no	Yes
**Sdcdgres**<br>length of time in Canada since immigration	1= Length of time in Canada since immigration 0 - 9 years<br>2= Length of time in Canada since immigration 10 - 121 years		1= < 10 years<br>2= ≥ 10 years	Yes
**sdcdglhm**<br>Language(s) spoken most often at home	1= English (with or without language other than French)<br>2= French (with or without language other than English)<br>3= English and French (with or without other language)<br>4= Neither English nor French (Other)		1= English<br>2= French<br>3= English and French<br>4= Other	Yes
**dhh_own**<br>Dwelling ownership- own or rent	1= Owned by member of hhld, even if it is still being paid for<br>2= Rented, even if no cash rent is paid		1= Owned<br>2= Rented	Yes
**sdc_015**<br>Aboriginal identity-First nations/Metis/ Inuit	1= Yes<br><br>2= No		1= Yes<br><br>2= No	Yes

**ehg2dvh3**<br>Highest level of education-household	1= Less than secondary school graduation<br>2= Secondary school graduation, no post-secondary education<br>3= Post-secondary certificate diploma or univ degree		1= ≤ secondary school graduation<br>2= > secondary school graduation	Yes
**lbfdvpft** and **mac_010** related to employment		Employment status	1= Full-time employed (lbfdvpft==1)<br>2= Part-time employed (lbfdvpft==2)<br>3= Unemployed (mac_010==2)	Yes
**sdcdgcgt**<br>Cultural/racial background	1= White<br>2= Non-white		1= White<br>2= Non-white	Yes
**2. General or Mental Health related variables**				
**gen_020**<br>Perceived life stress	1= Not at all stressful<br>2= Not very stressful<br>3= A bit stressful<br>4= Quite a bit stressful<br>5= Extremely stressful		1= not stressful<br>2= stressful (including extremely stressful)	Yes
**gen_030**<br>Sense of belonging to local community	1= Very strong<br>2= Somewhat strong<br>3= Somewhat weak<br>4= Very weak		1= Strong (1 or 2)<br>2= Weak (3 or 4)	Yes
**gen_015**<br>Perceived mental health	1= Excellent<br>2= Very good<br>3= Good<br>4= Fair<br>5= Poor		1= Good to excellent<br>2= Fair to poor	Yes
**gen_005**<br>Perceived general health	1= Excellent<br>2= Very good<br>3= Good<br>4= Fair<br>5= Poor		1= Good to excellent<br>2= Fair to poor	Yes
**3. Dental Health related variables**				

**den_005**<br>Has at least one of own teeth	1= yes<br>2= no		1= yes<br>2= no	Yes
**den_010**<br>Frequency of tooth brushing	Continuous variable		1= less than 2 times<br>2= two times or more	Yes
**den_020**<br>Wears dentures/ false teeth	1= yes<br>2= no		1= yes<br>2= no	Yes
**den_030**<br>How often do you usually see a dental professional	1 = More than once a year for check-ups or treatment<br>2 = About once a year (for check-ups or treatment)<br>3 = Less than once a year (for check-ups or treatment)<br>4 = Only for emergency care<br>5 = Never		1 = More than once a year for check-ups or treatment<br>2 = Other	Yes
**den_035**<br>Last time visited a dental professional	1= Less than 1 year to 1 year ago<br>2= More than 1 year to 2 years ago<br>3= More than 2 years to 3 years ago<br>4= More than 3 years to 4 years ago<br>5= More than 4 years to 5 years ago<br>6= More than 5 years ago		1= one year or less<br>2= more than one year	Yes
**den_040**<br>Avoid going to dental professional because of the cost	1= yes<br>2= no		1= yes<br>2= no	Yes
**oht_005**<br>Perceived oral health	1= Excellent<br>2= Very good<br>3= Good<br>4= Fair<br>5= Poor		1= Good to excellent<br>2= Fair to poor	Yes
**oht_010**<br>Satisfaction with teeth/denture appearance	1= Very satisfied<br>2= Satisfied<br>3= Neither satisfied nor dissatisfied		1= Dissatisfied and very dissatisfied<br>2= Neither satisfied nor dissatisfied	Yes

	4= Dissatisfied<br>5= Very dissatisfied		3= Satisfied and very satisfied	
**oht_015**<br>Problem with mouth-uncomfortable to eat food-freq-12 mo	1= Often<br>2= Sometimes<br>3= Rarely<br>4= Never		1= Rarely/never<br>2= Often/sometimes	Yes
**oht_020**<br>Problem with mouth-avoid particular food-freq-12 mo	1= Often<br>2= Sometimes<br>3= Rarely<br>4= Never		1= Rarely/never<br>2= Often/sometimes	Yes
**oht_025**<br>Problem with mouth-other persistent pain-freq-12 mo	1= Often<br>2= Sometimes<br>3= Rarely<br>4= Never		1= Rarely/never<br>2= Often/sometimes	Yes
**oht_040**<br>Had bleeding gums-frequency	1= Often<br>2= Sometimes<br>3= Rarely<br>4= Never		1= Rarely/never<br>2= Often/sometimes	Yes
**oht_050**<br>Had persistent bad breath-frequency (past 12mo)	1= Often<br>2= Sometimes<br>3= Rarely<br>4= Never		1= Rarely/never<br>2= Often/sometimes	Yes
**Dependent variable**		dental insurance	1= Employment-based insurance (den_050a==1)<br>2= Government-based insurance (den_050b==1 + den_050d ==1 + den_050e ==1)<br>3= Private insurance (den_050c==1)<br>4= No insurance (den_045 ==2)	Yes
