# Supplementary material for: Socio-demographic predictors of not having private dental insurance coverage: machine-learning algorithms may help identify the disadvantaged
Source: BMC Public Health. 2024 May 23;24:1386. doi: 10.1186/s12889-024-18868-1 (PMC11112852; doi:10.1186/s12889-024-18868-1)
Supplement: Supplementary file 2 — Supplementary Material 2 [file 12889_2024_18868_MOESM2_ESM.pdf]

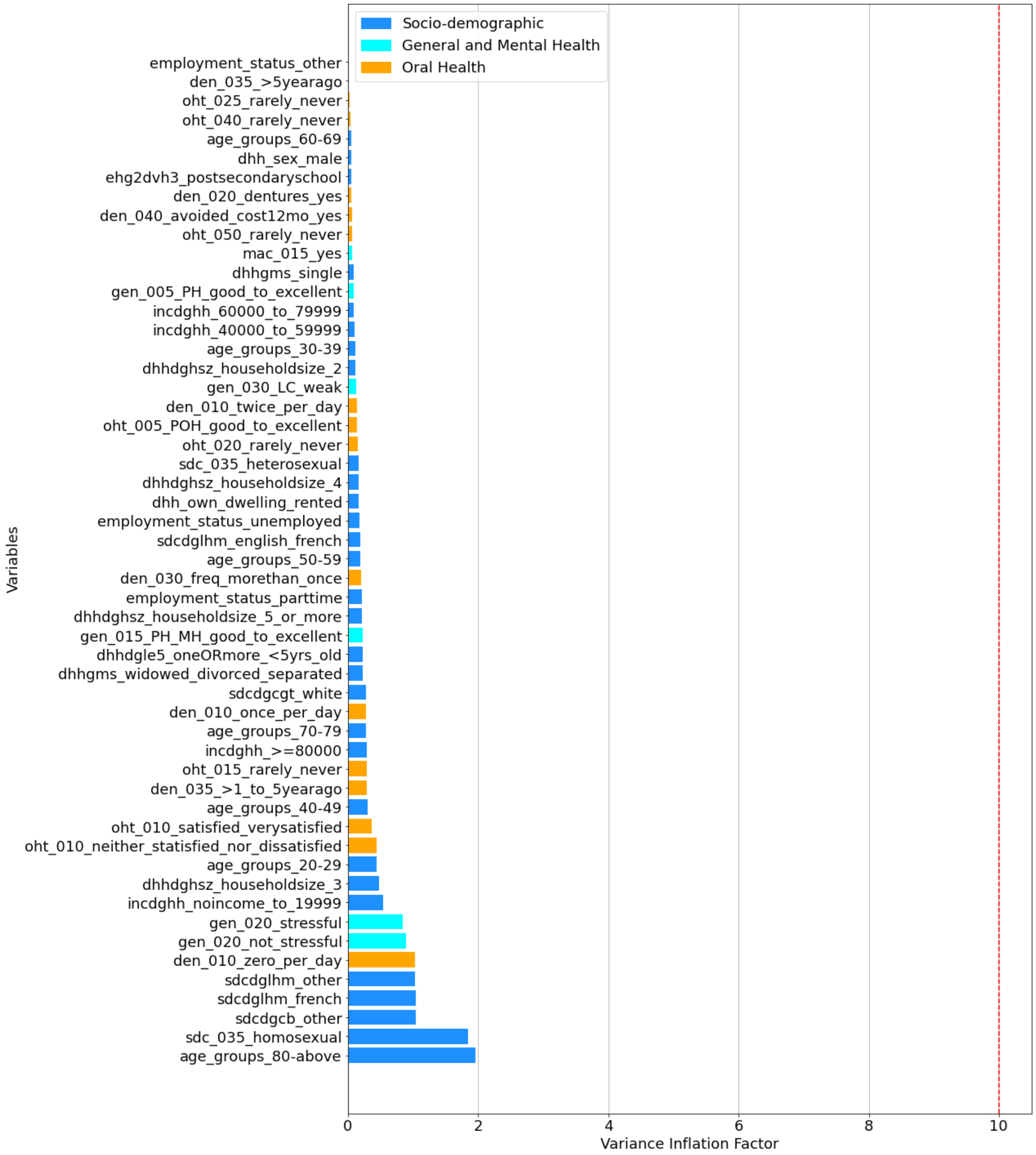

Figure S1. Selected independent variables (Features) based on the Variance Inflation Factor (VIF) analysis. Multicollinearity occurs when independent variables are highly correlated with each other, which can make it difficult to determine the individual impact of each predictor on the dependent variable. We have implemented the Variance Inflation Factor (VIF) analysis to detect the multicollinearity amongst all variables, where a VIF ≥ 5 indicates potential problematic levels of multicollinearity and VIF ≥ 10 indicates extreme multicollinearity.
