# Supplementary material for: Socio-demographic predictors of not having private dental insurance coverage: machine-learning algorithms may help identify the disadvantaged
Source: BMC Public Health. 2024 May 23;24:1386. doi: 10.1186/s12889-024-18868-1 (PMC11112852; doi:10.1186/s12889-024-18868-1)
Supplement: Supplementary file 3 — Supplementary Material 3 [file 12889_2024_18868_MOESM3_ESM.pdf]

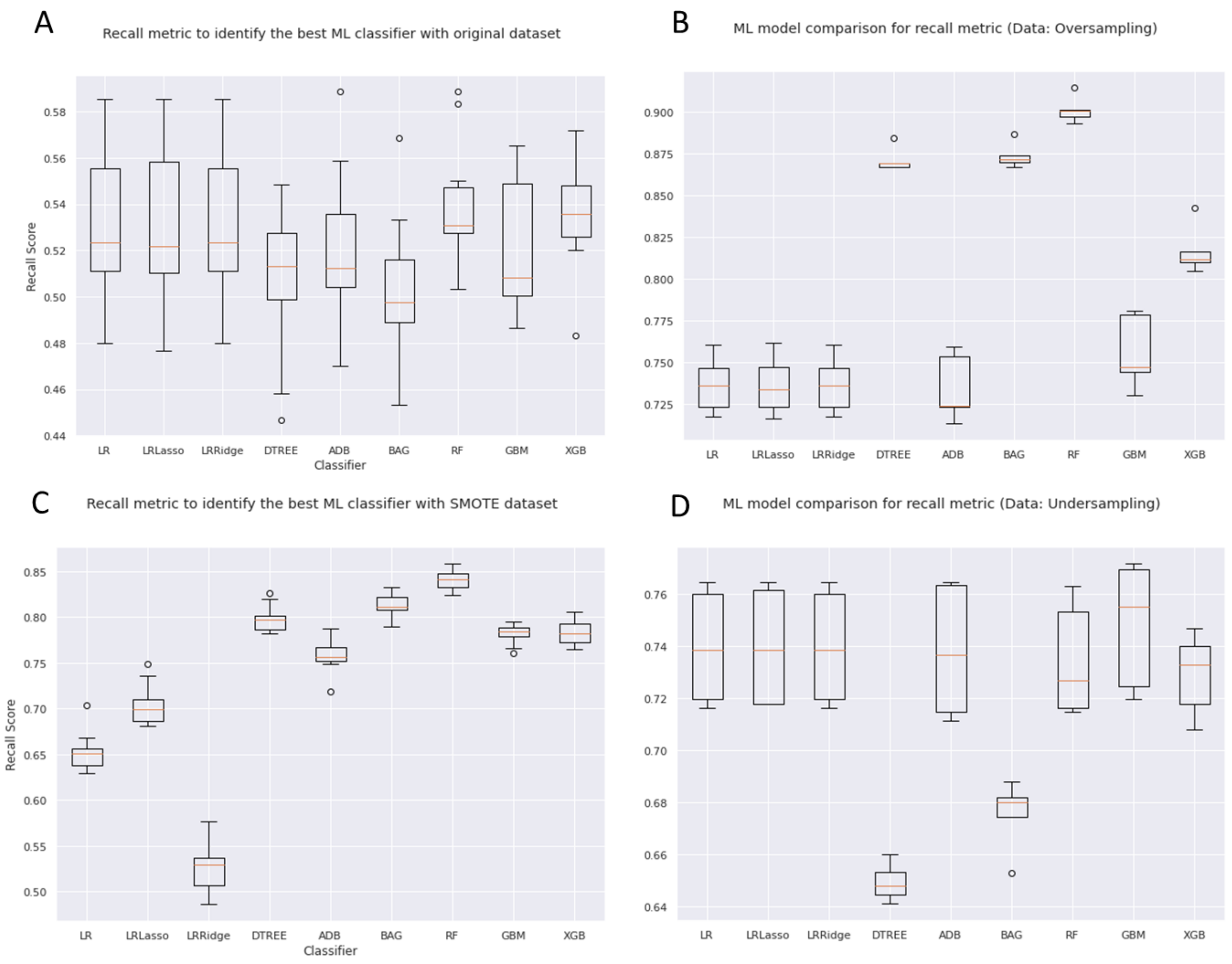

Figure S2. Performance of nine different supervised machine learning (ML) models on original dataset and three datasets generated from resampling techniques. Recall of nine supervised ML algorithms were compared in their ability to discriminate between not having private dental insurance and having private dental insurance.

Random Forest (RF) was the top performer with the original dataset (recall: 53.88%), the SMOTE dataset (recall: 84.07%), and Oversampling dataset (recall: 89.31%), and **Gradient Boosting** (GBM) with the Undersampling dataset (recall: 74.30%). These models were chosen in subsequent modelling and analysis.

LR, logistic regression; LASSO, least absolute shrinkage and selection operator; DTREE, decision tree; BAG, bootstrap aggregating; RF, random forest; GBM, gradient boosting machines; ADB, adaptive boosting; and XGB, XGBoost.
